# Commercially available mobile health applications for obesity across the lifespan: a systematic search and quality assessment

**DOI:** 10.1038/s41366-026-02097-0

**Published:** 2026-05-05

**Authors:** Lena Sophia Steubl, Marie von Altenbockum, Alexandra Portenhauser, Laura Simon, Michael Stach, Yannik Terhorst, Stephanie Brandt-Heunemann, Rüdiger Pryss, Martin Wabitsch, Harald Baumeister

**Affiliations:** 1 Department of Clinical Psychology and Psychotherapy, Ulm University, Ulm, Germany; 2 German Center for Child and Adolescent Health (DZKJ), Partner Site Ulm, Ulm, Germany; 3 German Center for Mental Health (DZPG), Partner Site Ulm, Ulm, Germany; 4 Institute for Clinical Epidemiology and Biometry, University of Würzburg, Würzburg, Germany; 5 Department of Psychology, Ludwig-Maximilians-Universität Munich, Munich, Germany; 6 German Center for Mental Health (DZPG), Partner Site Munich, Munich, Germany; 7 Division of Paediatric Endocrinology and Diabetology, University Hospital for Paediatrics and Adolescent Medicine Ulm, Ulm, Germany

**Keywords:** Weight management, Translational research, Paediatrics

## Abstract

**Background:**

Mobile health applications (MHAs) represent a promising low-threshold tool to support obesity treatment. While commercially available MHAs may be most accessible to potential users, concerns exist regarding their quality, data protection, and evidence base. Therefore, this study aimed to systematically identify and evaluate these aspects.

**Methods:**

A systematic search was conducted in the Apple App Store and Google Play Store, identifying 1220 apps. After a two-stage screening process, *n* = 21 MHAs met the inclusion criteria and were evaluated independently by two raters using the German version of the Mobile App Rating Scale (MARS-G) with the five subscales Engagement, Functionality, Esthetics, Information, and Therapeutic Gain. Additionally, data on general characteristics (including information on the age group targeted and data protection and safety measures), inclusion of established treatment components, and evidence base, were collected.

**Results:**

None of the included MHAs explicitly targeted children or adolescents. Concerning privacy and safety, notable deficiencies were identified, particularly with regard to the absence of active confirmation of privacy policy and/or terms of service and a lack of integrated emergency features. Included MHAs demonstrated moderate overall quality (*M* = 3.31, SD = 0.50). The lowest ratings were observed on the subscales Information (*M* = 2.74, SD = 0.65) and Therapeutic Gain (*M* = 2.39, SD = 0.70). Inclusion of all components of evidence-based obesity treatment guidelines was found in only *n* = 5 MHAs (23.8%). Published evidence for effectiveness was identified for only *n* = 2 MHAs (9.5%).

**Conclusions:**

The findings indicate a moderate quality level of commercially available MHAs for obesity treatment, with significant deficits in data protection and safety, content, therapeutic value, inclusion of established treatment components, and scientific evidence. To support safe and effective care for those affected by obesity, there is a need for further research and joint efforts (e.g., in terms of translation into routine practice).

## Introduction

Obesity is a major global health concern, affecting approximately 640 million adults and 110 million children and adolescents worldwide and accounting for more than 1.2 million deaths annually in the WHO European Region alone [1]. The disease is not merely the result of lifestyle choices, but of complex interactions among genetic, neurobiological, behavioral, and socioeconomic factors [2–4]. For example, dysregulated appetite and reward signaling, particularly involving leptin, dopamine, and the hypothalamic circuitry, contribute to persistent overeating [5–7]. Moreover, social determinants such as low income, reduced health literacy, and chronic psychosocial stress further increase the risk of developing obesity and complicate its management [2, 8, 9]. The disease is a leading risk factor for type 2 diabetes, cardiovascular diseases, as well as certain cancers, and is associated with a range of comorbid conditions (e.g., osteoarthritis) [10, 11]. Stigmatizing attitudes toward people with obesity remain widespread and are associated with reduced self-esteem, internalized weight stigma, anxiety, depressive symptoms, and even suicidal behaviors [12–15]. Obesity often begins early in life, and pediatric obesity typically persists into adulthood and is already associated with metabolic, physical, and psychosocial complications [16–18]. Therefore, early identification and intervention are critical, not only during adulthood but across the entire lifespan.

Treatment options are threefold: lifestyle modification (i.e., dietary modification, regular exercise, and behavior therapy), pharmacotherapy, and bariatric surgery [19, 20]. Behavioral strategies, particularly dietary and physical activity modifications, are the first-line treatment and represent the foundation of care. Pharmacotherapy and bariatric surgery are considered in selected cases and may enhance outcomes when combined with lifestyle interventions [19, 20].

Despite these existing treatments, long-term weight maintenance remains a major challenge, and stigma-related barriers, such as feelings of shame and experiences of discrimination, as well as major structural barriers to care, frequently prevent individuals living with obesity from seeking help [12, 19, 21, 22].

Digital health interventions may help overcome these barriers and ensure long-term support by providing low-threshold and anonymous assistance, either as stand-alone tools or as part of combined treatment models (i.e., blended care, where digital components are integrated with face-to-face treatment) [23, 24]. In this context, two main forms of digital health interventions are typically distinguished: video-based treatment and Internet- and mobile-based interventions (IMIs) [25]. While synchronously provided video-based treatment increases accessibility—particularly for individuals in rural areas or with mobility limitations—it does not expand treatment capacity and might not address stigma-related barriers [25]. In contrast, IMIs offer scalable and flexible formats that can reach large populations [25]. They can be delivered app-based (i.e., mobile health applications; MHAs) or web-based and with or without guidance (i.e., support provided during the use of IMIs, e.g., regular feedback) [25]. Their content typically includes psychoeducation, self-monitoring tools, goal setting, or behavior change techniques [25, 26]. Meta-analytical evidence has demonstrated the effectiveness of IMIs across various mental and somatic health conditions (e.g., chronic pain, diabetes, depression, anxiety) for both youth and adult populations [27–32]. They have also been shown to support obesity treatment, especially when delivered in guided formats: studies report clinically relevant weight loss and improvements in self-regulation [26, 33–36]. In particular, a meta-analysis found no differences between web-based and offline interventions regarding weight and body mass index (BMI) changes [37]. Web-based interventions even led to a greater short-term weight loss [37].

Despite this promising evidence, the IMIs examined in the aforementioned studies are typically not the ones most readily available to users. Outside of regulated products such as the Digital Health Applications (*Digitale Gesundheitsanwendungen*, DiGAs) available in Germany [25], individuals who seek support are more likely to encounter commercially available MHAs in app stores, which are usually not scientifically evaluated. Those MHAs often show deficits in content quality, safety features, or therapeutic value, as demonstrated by evidence from other health domains [38–41]. Additionally, user ratings, which often guide potential users’ choices, are typically based on usability rather than clinical effectiveness or content quality [42, 43].

Against this background, a systematic search and quality assessment of commercially available MHAs for obesity treatment seems worthwhile in order to determine whether the previously observed quality deficits of commercially available MHAs also apply to those targeting obesity. Stapelfeldt et al. already conducted such a search and quality rating based on the German version of the Mobile App Rating Scale (MARS-G) [38, 44, 45]. The *n* = 10 MHAs included met some guideline recommendations and were rated to be of adequate to good quality [38]. However, their review was restricted to free, German-language apps available in both major app stores and did not employ automated search tools such as web scrapers. Moreover, age-related aspects were not considered, leaving unanswered whether commercially available MHAs adequately address the needs of different age groups.

The present review builds on and extends this work by adopting a broader, lifespan-oriented perspective and employing an international, web-scraper-assisted search strategy. This approach enables the systematic identification of potential gaps in tools for children and adolescents and provides a more comprehensive view of the current landscape of digital obesity support.

The aim of this study is therefore to provide an overview of the quality and characteristics of commercially available MHAs for obesity. The collected data comprise general app characteristics (including targeted age groups and information on data security and data protection), MHA quality ratings, the inclusion of established treatment components, and the presence of a scientific evidence base. The results are intended to be integrated into a publicly accessible platform (i.e., Mobile Health App Database (MHAD); www.mhad.science) to support potential users in making informed decisions and to improve transparency regarding available MHAs [46].

## Methods

### App identification

A systematic search of commercially available MHAs for obesity was conducted in November 2024 in the two major European app stores Apple App Store and Google Play Store, using a web scraper developed for MHAD [46]. This procedure has been successfully employed in previous studies [39, 40, 47, 48]. A predefined list of 36 German and English obesity-related search terms was used (e.g., “obesity”, “overweight”, “weight management”, and corresponding German terms; see Supplementary Material Table A for the full list), with each term entered individually, as combined or nested queries are not supported in both app stores’ search algorithms. The terms were selected based on their relevance and likelihood of being used by individuals seeking app-based obesity treatment and reviewed by an expert in the field of obesity (MW).

### Screening procedures (Level 1 and Level 2)

The resulting apps underwent a two-stage screening process based on predefined inclusion and exclusion criteria. During the Level 1 screening, inclusion was based on information provided in the app store descriptions. Apps were included if they (1) were directed at individuals with obesity or their caregivers, (2) centrally targeted obesity treatment or mentioned weight reduction as a main goal, and (3) were available in German or English. In the Level 2 screening, the remaining apps were downloaded and tested on either an Apple iPhone 15 (Apple Inc., Cupertino, CA, USA) or a Samsung Galaxy S23 (Samsung Electronics Co., Ltd., Suwon, South Korea), depending on the respective app store. Apps were excluded if they (1) addressed other conditions or did not include at least one of the core modules of obesity treatment, (2) were designed solely for healthcare professionals, (3) were available only in blended care formats, (4) functioned only as BMI calculators or tracking tools, (5) required external devices (e.g., smartwatch), (6) were tablet- or computer-only, (7) were duplicates, or (8) required access codes or login credentials restricted to specific users, such as members of certain clinics or institutions and for which access could not be obtained. Developers of apps requiring those access codes or login credentials were contacted to request access. If no response was received within 4 weeks, the app was excluded. Only MHAs functioning on the respective test devices were included in the final sample.

### Data extraction of general characteristics

To extract data of included MHAs, an adapted version of the classification page of the MARS-G (see “Quality assessment”) was used [44]. General information extracted included: (1) name, (2) platform (i.e., Apple App Store or Google Play Store), (3) user rating (i.e., star rating and number of ratings), (4) developer, (5) age group (as stated in the app store description), (6) specifically targeting children/adolescents (yes/no), (7) associated costs (yearly), (8) affiliation (i.e., unknown, commercial, university, non-governmental organization, government), (9) certified (i.e., have undergone a formal regulatory approval process in their respective country; yes/no), and (10) employed methods. Additionally, information on data security and data protection was extracted (yes/no): (1) allows password protection, (2) requires login, (3) provides privacy policy, (4) requires active confirmation of privacy policy and/or terms of service during onboarding, (5) provides information about data handling, (6) provides information about funding/conflicts of interest, (7) provides contact information, (8) indicates security of data transmission, (9) includes emergency functions, and (10) provides data protection strategies in case of phone loss. All information was extracted by one reviewer (MA).

### Quality assessment

All included MHAs were then evaluated by two independent raters (MA and MG) using the German version of the Mobile App Rating Scale (MARS-G) [44], which is based on the original MARS [45]. Both raters completed the recommended training modules [44, 45] and received additional supervision from two experienced clinical psychologists (LSS and LS; MSc, advanced clinical training).

The MARS-G includes four main subscales (Engagement with five items, Functionality with four items, Esthetics with three items, and Information with seven items). Contrary to the original MARS, the MARS-G includes the additional subscale Therapeutic Gain with four items (i.e., benefit for patients, benefit for therapists, security [renamed from “risk” to clarify coding directions], transferability to routine care). All items are rated on a five-point Likert scale (1 = inadequate, 2 = poor, 3 = acceptable, 4 = good, and 5 = excellent). The scale has demonstrated acceptable to excellent internal consistency on all four main subscales (*ω* = 0.74–0.91) [44]. Correlation coefficients between corresponding scales of MARS-G and MARS range between 0.93 and 0.98 [44].

Prior to rating, each MHA was tested for a minimum of 15 min and explored for as long as necessary to assess its core content and functionalities. If available in both the Apple App Store and Google Play Store, both MHAs were assessed separately. Discrepancies between raters—defined as a difference of two or more points on any item—were resolved through discussion.

### Inclusion of established treatment components

In addition, each MHA was assessed for inclusion of treatment components following established clinical guidelines for obesity treatment [20, 49, 50], which identify lifestyle modification (i.e., dietary modification, physical activity, and behavioral strategies) as the foundation and first-line treatment of obesity. Accordingly, the presence of the following evidence-based components was recorded: (1) nutrition counseling, (2) content related to physical activity, and (3) behavioral strategies, including elements such as emotion regulation and motivation enhancement. All information was extracted by a single reviewer (MA).

### Evidence base

To evaluate the scientific evidence base of the included MHAs, both the MHAs themselves and associated sources—such as official websites and app store entries—were screened for references to peer-reviewed publications on the effectiveness of the respective MHA. Only studies published in scientific journals were considered. This approach aligns with Item 7 of the MARS-G Information subscale, which explicitly assesses the presence of scientific evaluation [44]. This information was again extracted by a single reviewer (MA).

### Data analysis

All analyses were conducted in R [51]. First, to ensure quality of ratings, inter-rater reliability between the two raters across the four main subscales (Engagement, Functionality, Esthetics, Information) was assessed using intraclass correlation coefficients (ICC) based on a two-way mixed-effects model with absolute agreement. ICC values were interpreted as follows: ICC < 0.50 = poor, 0.50 – 0.75 = moderate, 0.75–0.90 = good, >0.90 = excellent [52].

For all subsequent analyses, scores from both raters were averaged at the item level. For each MHA, mean scores were calculated for the four main subscales and subsequently averaged to compute an overall MHA quality score. Across all included MHAs, main subscale and overall quality means as well as standard deviations were calculated. Overall quality scores on both individual MHA level and across all included MHAs were categorized as low (<2.5), moderate (2.5–4.0), or high (>4.0) following other MARS-G studies [39, 40].

To assess the validity of user star ratings, Pearson correlations between those and MARS-G quality scores (total and main subscales) were computed. Additionally, a Wilcoxon rank-sum test was performed to test for differences in total quality scores between MHAs from the Apple App Store and Google Play Store.

The additional MARS-G subscale Therapeutic Gain was analyzed separately. For each MHA, a mean score was calculated, and both item and subscale scores were again categorized as low (<2.5), moderate (2.5–4.0), or high (>4.0) quality. This classification was again made in accordance with other MARS-G studies [39, 40].

General characteristics (including information on age group and data security and data protection), inclusion of established treatment components, and evidence base of included MHAs were synthesized descriptively.

### Ethics approval and consent to participate

This study did not involve human participants, human data, or animal subjects. Therefore, ethical approval and informed consent were not required. All methods were carried out in accordance with relevant guidelines and regulations.

## Results

### MHA identification

**Fig. 1:**
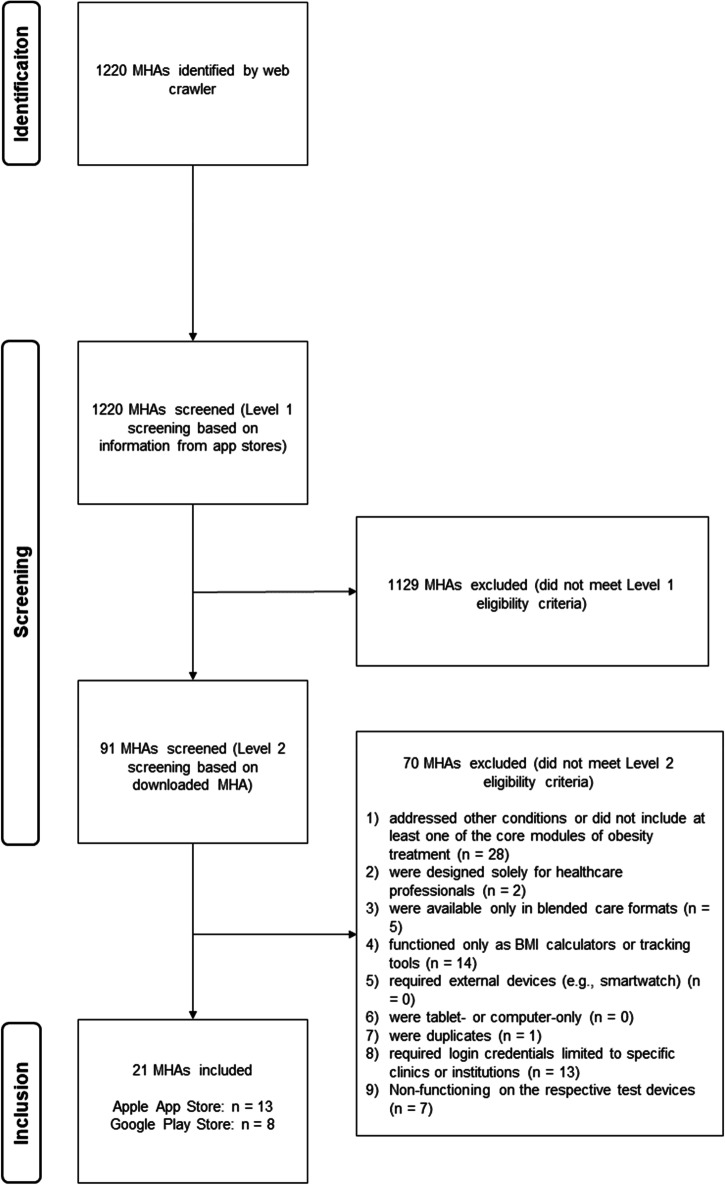
Flowchart of the MHA selection process. The figure illustrates the identification, screening, and inclusion of MHAs.

A total of 1220 apps were identified through a systematic app store search in November 2024. After applying the two-stage screening procedure, *n* = 21 MHAs (1.7%) were included in the quality assessment. Of note, these entries corresponded to *n* = 18 unique MHAs, as three MHAs were listed in both the Apple App Store and the Google Play Store. Of the *n* = 21 included MHAs, *n* = 13 (61.9%) were available in the Apple App Store and *n* = 8 (38.1%) in the Google Play Store. The process of MHA identification is detailed in the flow chart (Fig. 1).

### General characteristics

User star ratings of the included MHAs ranged from 3.7 to 5.0 (*M* = 4.41, SD = 0.42). Annual costs varied between 0€ and 399.99€ (*M* = 97.20, SD = 111.20). Most MHAs were affiliated with commercial providers (*n* = 15; 71.4%), followed by unknown affiliations (*n* = 5; 23.8%), and university institutions (*n* = 1; 4.8%). Only *n* = 2 MHAs (9.5%) had undergone a formal regulatory approval process, both were German Digital Health Applications (DiGAs). All MHAs contained advice (*n* = 21; 100%). Most included informational/educational content (*n* = 18; 85.7%) and monitoring/tracking features such as weight or activity logs (*n* = 20; 95.2%). Additionally employed features included reminders/reinforcers (*n* = 17; 81.0%), data collection tools (*n* = 15; 71.4%), goal tracking content (*n* = 13; 61.9%), gamification elements (*n* = 9; 42.9%), physical exercises (*n* = 5; 23.8%), strategies/skills training (*n* = 4; 19.0%), feedback functions (*n* = 2; 9.5%), and relaxation exercises (*n* = 1; 4.8%). Detailed characteristics are displayed in Table 1.

**Table 1:** General characteristics of included MHAs (*n* = 21) in descending order of the total quality mean score.

Name	Platform	User star rating (ratings total)	Developer	Age group^a^	Specifically targeting children/adolescents	Associated costs (yearly)	Affiliation	Certified^b^	Employed methods
Oviva Direkt: Gesund Abnehmen	Android	4.5 (8727)	Oviva AG	All ages	No	0€	Commercial	Yes	- Data collection - Monitoring/tracking - Information/education - Advice - Goal tracking - Strategies/skills training - Physical exercises - Gamification - Reminders/reinforcers
Oviva Direkt: Gesund Abnehmen	iOS	4.5 (4638)	Oviva AG	16 years +	No	0€	Commercial	Yes	- Data collection - Monitoring/tracking - Information/education - Advice - Goal tracking - Strategies/skills training - Physical exercises - Gamification - Reminders/reinforcers
MYNDSET	iOS	– (0)	MYND Therapeutics Inc.	16 years +	No	399.99€	Commercial	No	- Data collection - Monitoring/tracking - Feedback - Information/education - Advice - Goal tracking - Strategies/skills training - Gamification - Reminders/reinforcers
Simple: Weight Loss Coach	Android	4.1 (94 200)	Simple.Life Apps Inc	All ages	No	139.96€	Commercial	No	- Data collection - Monitoring/tracking - Information/education - Advice - Goal tracking - Gamification - Reminders/reinforcers
Valea: Essensplan zum Abnehmen	iOS	4.6 (80)	SevenCooks GmbH & Co. KG	9 years +	No	71.99€	Commercial	No	- Monitoring/tracking - Information/education - Advice - Goal tracking - Strategies/skills training - Reminders/reinforcers
Weight Watchers Programm	Android	3.7 (591 000)	WW International, Inc.	All ages	No	199.99€	Commercial	No	- Data collection - Monitoring/tracking - Information/education - Advice - Goal tracking - Gamification - Reminders/reinforcers
Simple: dein Ernährungshelfer	iOS	4.5 (7359)	AM APPS	18 years +	No	139.96€	Commercial	No	- Data collection - Monitoring/tracking - Information/education - Advice - Goal tracking - Gamification - Reminders/reinforcers
Weight Watchers Programm	iOS	4.5 (94 495)	WW International, Inc.	18 years +	No	199.99€	Commercial	No	- Data collection - Monitoring/tracking - Information/education - Advice - Goal tracking - Gamification - Reminders/reinforcers
The Fast 800	Android	4.7 (23)	The Fast 800	All ages	No	289.99 €	Commercial	No	- Data collection - Monitoring/tracking - Information/education - Advice - Goal tracking - Relaxation exercises - Physical exercises - Reminders/reinforcers
Gesünder leben: myFoodDoctor	iOS	3.8 (1007)	FoodDoc GmbH	9 years +	No	89.99€	Commercial	No	- Monitoring/tracking - Feedback - Information/education - Advice - Reminders/reinforcers
Budiyu	iOS	5 (1)	Stitching HappiApp	13 years + (parents of kids aged 6–12 years)	No (but their parents)	0€	Commercial	No	- Monitoring/tracking - Information/education - Advice - Reminders/reinforcers
LeanOnMe	iOS	5 (4)	Loopcare GmbH	18 years +	No	239.99€	Commercial	No	- Data collection - Monitoring/tracking - Information/education - Advice - Goal tracking - Gamification - reminders/reinforcers
Low Fat Diet Recipes App	Android	4.2 (95)	Riafy Technologies	All ages	No	25.98€	Unknown	No	- Monitoring/tracking - Advice - Physical exercises
Abnehmen mit Freunden	iOS	4.4 (124)	Social First Ltd.	4 years +	No	0€	Commercial	No	- Data collection - Monitoring/tracking - Information/education - Advice - Goal tracking - Reminders/reinforcers
INSELhealth – cardio fit	iOS	5 (1)	Skyscraper Software GmbH	16 years +	No	0€	University	No	- Monitoring/tracking - Information/education - Advice - Goal tracking - Reminders/reinforcers
BMI Calculator ~	iOS	4.7 (19)	LIFE+	4 years +	No	103.48€	Unknown	No	- Data collection - Monitoring/tracking - Advice - Goal tracking - Reminders/reinforcers
Lose weight with no stress	Android	4.9 (5 130)	Opti-fit	All ages	No	19.99€	Unknown	No	- Data collection - Monitoring/tracking - Information/education - Advice - Reminders/reinforcers
Barilifestyle	Android	– (0)	OL-fitness	All ages	No	109.99€	Commercial	No	- Data collection - Monitoring/tracking - Advice - Physical exercises
Dr.Cook	iOS	4.5 (41)	MediCook	13 years +	No	9.99€	Commercial	No	- Monitoring/tracking - Information/education - Advice - Gamification - Reminders/reinforcers
OBEASIGO	iOS	– (0)	Phantom smart labs	Not specified	No	0€	Unknown	No	- Data collection - Information/education - Advice
BMI Calculator Weight Tracker	Android	3.8 (13 800)	EZHIL	All ages	No	0€	Unknown	No	- Data collection - Monitoring/tracking - Advice

^a^As stated in the app store description.

^b^MHAs that had undergone a formal regulatory approval process in their respective country. In Germany, this corresponds to Digital Health Applications (*Digitale Gesundheitsanwendungen*, DiGAs), which can be prescribed within statutory health insurance.

### Age group

With regard to the intended age group stated in the app store descriptions, most MHAs were labeled for all ages (*n* = 7; 33.3%) or for adults, defined as users aged 18 years and older (*n* = 4; 19.0%). Additional minimum-age classifications included labels for use from 16 years (*n* = 3; 14.3%), from 13 years (*n* = 2; 9.5%), from 9 years (*n* = 2; 9.5%), and from 4 years (*n* = 2; 9.5%). For *n* = 1 MHA (4.8%), age-related information was not available.

None of the MHAs (0.0%) explicitly targeted children or adolescents. However, *n* = 1 MHA (4.8%) mentioned potential use by parents of children aged 6–12 years with genetic forms of obesity.

### Information on data security and data protection

Regarding data security and data protection features, most MHAs provided privacy policies (*n* = 17; 81.0%), included contact information (*n* = 16; 76.2%), provided information about data handling (*n* = 15; 71.4%), and offered data protection strategies in case of phone loss (*n* = 15; 71.4%). Password protection was present in *n* = 14 MHAs (66.7%), *n* = 12 MHAs (57.1%) indicated security of data transmission, and login was required in *n* = 11 MHAs (52.4%). Only *n* = 10 MHAs (47.6%) required active confirmation of privacy policy and/or terms of service and *n* = 1 MHA (4.8%) provided emergency functions. No MHA (0.0%) disclosed information about funding or conflicts of interest. Detailed information on data security and data protection is displayed in Table 2.

**Table 2:** Information on data security and data protection of included MHAs (*n* = 21) in descending order of the total quality mean score.

Name	Platform	Password protection	Login	Privacy policy	Active confirmation of privacy policy and/or terms of service	Information about data handling	Information about funding/conflicts of interest	Contact information	Security of data transmission	Emergency functions	Data protection strategies in case of phone loss
Oviva Direkt: Gesund Abnehmen	Android	X	X	X	X	X		X	X		X
Oviva Direkt: Gesund Abnehmen	iOS	X	X	X	X	X		X	X		X
MYNDSET	iOS	X	X	X		X		X			X
Simple: Weight Loss Coach	Android	X		X	X	X		X	X		X
Valea: Essensplan zum Abnehmen	iOS	X		X	X	X		X	X		X
Weight Watchers Programm	Android	X		X	X	X		X			X
Simple: dein Ernährungshelfer	iOS	X	X	X	X	X		X	X		X
Weight Watchers Programm	iOS	X	X	X	X	X		X			X
The Fast 800	Android	X	X	X	X	X		X	X		X
Gesünder leben: myFoodDoctor	iOS	X	X	X	X	X		X	X		X
Budiyu	iOS	X	X	X	X	X		X	X		X
LeanOnMe	iOS	X	X	X	X	X		X	X	X	X
Low Fat Diet Recipes App	Android							X			
Abnehmen mit Freunden	iOS		X	X		X		X			X
INSELhealth – cardio fit	iOS			X		X			X		
BMI Calculator ~	iOS							X			
Lose weight with no stress	Android										
Barilifestyle	Android	X	X	X		X		X	X		
Dr.Cook	iOS	X		X							
OBEASIGO	iOS										X
BMI Calculator Weight Tracker	Android			X					X		

### Quality assessment

There were no relevant discrepancies between raters that had to be resolved through discussion. Inter-rater reliability for the overall MARS score across the four original MARS subscales was good (ICC = 0.78; 95% CI [0.73, 0.81]), with subscale ICCs ranging from 0.59 to 0.80.

Overall quality of included MHAs including the four main subscales was moderate (*M* = 3.31; SD = 0.50). Among the main subscales, Functionality was rated highest across all MHAs (*M* = 3.87; SD = 0.44), followed by Esthetics (*M* = 3.44; SD = 0.68), Engagement (*M* = 3.20; SD = 0.52), and Information (*M* = 2.74; SD = 0.65). Of all individual MHAs, *n* = 3 (14.3%) were rated as of high quality, *n* = 16 (76.2%) as of moderate quality, and *n* = 2 (9.5%) as of low quality. The best-rated MHA according to the overall quality score was “Oviva Direkt” (Android) with a score of 4.3 (high). Results from the MARS-G quality rating (four main subscales and overall quality score) are displayed in Table 3.

**Table 3:** MARS-G main subscale scores and overall scores of included MHAs (*n* = 21) in descending order of the total quality mean score.

Name	Platform	Engagement	Functionality	Esthetics	Information	Overall quality
Oviva Direkt: Gesund Abnehmen	Android	4.0	4.5	4.3	4.2	4.3
Oviva Direkt: Gesund Abnehmen	iOS	4.0	4.4	4.3	4.1	4.2
MYNDSET	iOS	3.8	4.1	4.3	3.6	4.0
Simple: Weight Loss Coach	Android	3.7	4.2	4.0	2.8	3.7
Valea: Essensplan zum Abnehmen	iOS	3.5	4.4	4.3	2.3	3.6
Weight Watchers Programm	Android	3.4	4.0	3.8	2.9	3.5
Simple: dein Ernährungshelfer	iOS	3.4	4.4	3.7	2.7	3.5
Weight Watchers Programm	iOS	3.4	4.0	3.8	2.6	3.5
The Fast 800	Android	3.4	4.1	3.5	2.9	3.5
Gesünder leben: myFoodDoctor	iOS	3.2	3.8	4.0	3.0	3.5
Budiyu	iOS	3.1	4.0	3.8	2.6	3.4
LeanOnMe	iOS	3.8	3.1	3.0	3.1	3.2
Low Fat Diet Recipes App	Android	3.2	3.4	3.5	2.2	3.1
Abnehmen mit Freunden	iOS	2.9	4.1	2.8	2.5	3.1
INSELhealth – cardio fit	iOS	2.8	3.6	3.0	3.0	3.1
BMI Calculator ~	iOS	2.5	4.1	3.2	2.2	3.0
Lose weight with no stress	Android	2.9	3.6	2.7	2.4	2.9
Barilifestyle	Android	2.9	3.6	2.5	2.6	2.9
Dr.Cook	iOS	2.8	3.2	2.7	2.4	2.8
OBEASIGO	iOS	2.1	3.1	2.5	1.9	2.4
BMI Calculator Weight Tracker	Android	2.4	3.4	2.3	1.6	2.4

No significant differences regarding overall quality were found between MHAs of the Apple App Store and of the Play Store (Wilcoxon rank-sum test: *W* = 55; *p* > 0.05; Apple *M* = 3.33, SD = 0.47 vs. Google *M* = 3.28, SD = 0.57). User star ratings did not correlate significantly with the overall quality score (*r* = −0.02; 95% CI [−0.48, 0.52]; *p* > 0.05), nor with any main subscale score (*r* range: −0.19 to 0.16; all *p* > 0.05).

The Therapeutic Gain subscale received the lowest overall ratings across all MARS-G subscales (*M* = 2.39; SD = 0.70). The majority of MHAs (*n* = 15; 71.4%) were rated as low in therapeutic benefit (<2.5), while *n* = 5 (23.8%) showed moderate and *n* = 2 (9.5%) high therapeutic potential. At the item level, only *n* = 2 MHAs (9.5%) received high scores for perceived patient benefit. None of the MHAs (0.0%) were rated highly for usefulness to therapists, *n* = 5 (23.8%) received high ratings for security, and *n* = 3 (14.3%) for transferability to routine care.

The item-level and mean scores of the Therapeutic Gain subscale for each MHA are presented in Table 4, ratings on the Therapeutic Gain subscale per item across all MHAs are displayed in Supplementary Material Fig. A.

**Table 4:** Item-level and overall subscale score of the Therapeutic Gain subscale of included MHAs (*n* = 21) in descending order of the total quality mean score.

Name	Platform	Benefit for patients	Benefit for therapists	Security	Transfer-ability to routine care	Overall
Oviva Direkt: Gesund Abnehmen	Android	high	moderate	high	high	***high***
Oviva Direkt: Gesund Abnehmen	iOS	high	moderate	high	high	***moderate***
MYNDSET	iOS	moderate	moderate	high	high	***moderate***
Simple: Weight Loss Coach	Android	moderate	moderate	moderate	low	***moderate***
Valea: Essensplan zum Abnehmen	iOS	moderate	low	moderate	low	***low***
Weight Watchers Programm	Android	moderate	low	moderate	low	***low***
Simple: dein Ernährungshelfer	iOS	moderate	moderate	moderate	low	***low***
Weight Watchers Programm	iOS	moderate	low	moderate	moderate	***moderate***
The Fast 800	Android	moderate	low	moderate	low	***low***
Gesünder leben: myFoodDoctor	iOS	moderate	low	moderate	low	***low***
Budiyu	iOS	moderate	low	moderate	low	***low***
LeanOnMe	iOS	moderate	moderate	high	low	***moderate***
Low Fat Diet Recipes App	Android	moderate	low	moderate	low	***low***
Abnehmen mit Freunden	iOS	low	low	moderate	low	***low***
INSELhealth – cardio fit	iOS	moderate	low	high	low	***moderate***
BMI Calculator ~	iOS	low	low	moderate	low	***low***
Lose weight with no stress	Android	moderate	low	moderate	low	***low***
Barilifestyle	Android	low	low	moderate	low	***low***
Dr. Cook	iOS	low	low	moderate	low	***low***
OBEASIGO	iOS	moderate	low	moderate	low	***low***
BMI Calculator Weight Tracker	Android	low	low	moderate	low	***low***

The item risk was renamed to security to clarify encoding directions. Scores are categorized as low (<2.5), moderate (2.5–4.0), or high (>4.0).

### Inclusion of established treatment components

Most MHAs included dietary advice or recipes (*n* = 17; 81.0%) and physical activity content (*n* = 14; 66.7%), but fewer addressed behavioral modification techniques (*n* = 8; 38.1%). Only *n* = 5 MHAs (23.8%) included components representing all three pillars of evidence-based obesity treatment.

An overview of treatment components included in each of the included MHAs is provided in Table 5.

**Table 5:** Inclusion of established treatment components in included MHAs (*n* = 21) in descending order of the total quality mean score.

Name	Platform	Dietary advice	Physical activity content	Behavioral strategies
Oviva Direkt: Gesund Abnehmen	Android	X	X	X
Oviva Direkt: Gesund Abnehmen	iOS	X	X	X
MYNDSET	iOS			X
Simple: Weight Loss Coach	Android	X	X	
Valea: Essensplan zum Abnehmen	iOS	X		X
Weight Watchers Programm	Android	X	X	
Simple: dein Ernährungshelfer	iOS	X	X	
Weight Watchers Programm	iOS	X	X	
The Fast 800	Android	X	X	X
Gesünder leben: myFoodDoctor	iOS	X		
Budiyu	iOS			X
LeanOnMe	iOS	X	X	X
Low Fat Diet Recipes App	Android	X	X	
Abnehmen mit Freunden	iOS	X	X	
INSELhealth – cardio fit	iOS		X	
BMI Calculator ~	iOS	X	X	
Lose weight with no stress	Android	X		
Barilifestyle	Android		X	
Dr.Cook	iOS	X		
OBEASIGO	iOS	X	X	X
BMI Calculator Weight Tracker	Android	X		

### Evidence base

Only two MHAs (9.5%) have undergone scientific evaluation. Both were the iOS and Android versions of Oviva Direkt, the only included certified MHAs. An RCT (*n* = 168) on the intervention reported a significantly greater weight loss in the intervention group (−3.2 kg ±3.2) compared to a waitlist control (−0.4 kg ±2.6; between-group effect of −2.9 kg; 95% CI [−3.8, −1.9]; *p* < 0.001) after 12 weeks, with positive results also for fat mass reduction, usability, and acceptability, but not for quality of life [53]. A retrospective study (*n* = 25,706) in a blended care context found average weight loss of 1.89 kg (SD = 7.82) at 1 month, increasing to 7.22 kg (SD = 9.67) after 12 months [54].

## Discussion

This systematic search and quality assessment provides a comprehensive overview of German- and English-language MHAs commercially available in major app stores (Apple App Store and Google Play Store) for the treatment of obesity. In total, *n* = 21 MHAs met the inclusion criteria—more than twice the number identified in the earlier review [38]—reflecting the broader, web-scraper-assisted search strategy and the inclusion of international MHAs. None of the included MHAs explicitly addressed children or adolescents. Data security and data protection features varied substantially, with several essential safeguards missing in many MHAs. On average, the included MHAs showed moderate quality ratings, with Functionality and Esthetics receiving the highest scores. Only a minority included all three pillars of evidence-based obesity treatment, and scientific evaluation was documented for just two MHAs, both certified.

Although several MHAs carried App Store age ratings such as 4 years or 9 years +, these thresholds reflect platform-specific minimum age recommendations rather than intentional pediatric design. In practice, none of the evaluated MHAs offered features, content, or guidance tailored to the developmental needs of children or adolescents, and only one MHA referenced potential use by parents of affected children. This highlights a substantial gap in the availability of evidence-based digital tools for younger age groups, despite the well-established importance of early intervention in pediatric obesity [16, 17]. However, it should be noted that while it is often assumed that children and adolescents may particularly benefit from IMIs due to their high affinity for technology, current evidence remains limited and suggests smaller effect sizes compared to adults [55, 56].

Nevertheless, digital tools may still represent a valuable complementary option. Yet, the potential benefit of such tools can only be realized if they meet fundamental standards of quality, safety, and data protection. The findings of this review indicate that these basic requirements are often not met. For example, less than half of the included MHAs required active confirmation of privacy policy and/or terms of service. This also echoes findings from similar studies [39, 40, 57, 58], suggesting widespread neglect of basic privacy standards. Moreover, prior studies have demonstrated that even MHAs which appear to be privacy-compliant often transmit user data to third parties without appropriate disclosure [59], thereby undermining user trust and violating fundamental data protection norms in the health sector [60]. Alarmingly, emergency functions were included in only one MHA, despite evidence of elevated suicide risk among individuals with obesity [15]. Taken together, these findings suggest that the already poor data protection ratings observed in this review may actually underestimate the severity of the issue.

The findings on the quality of included MHAs also align with previous evaluations both in the field and across different health domains employing similar methodologies [38–40, 57, 58]. The low engagement scores specifically reflect a commonly reported challenge of MHAs, the limited long-term user retention [61, 62]. A key contributing factor appears to be the lack of personalization and insufficient use of features known to enhance engagement, such as reminders, self-monitoring, and goal setting, which are essential for integration into users’ daily routines [63, 64]. Moreover, the low engagement ratings may also reflect a broader lack of individualization, which is particularly problematic given the heterogeneity of individuals affected by obesity, for instance with regard to age of onset, comorbidities, or socioeconomic background [21]. While sustained engagement is critical for (long-term) effectiveness [65], it may, however, not be prioritized by developers focused primarily on short-term metrics such as downloads or visibility. Therefore, beyond expert ratings of engagement, the availability of objective usage indicators (e.g., retention, completion, frequency of use) would help contextualize the real-world impact of MHAs, particularly when such interventions are implemented within healthcare systems or recommended in clinical contexts. At the same time, defining and measuring meaningful engagement remains challenging, as commonly reported usage metrics (e.g., logins or raw interaction counts) may not adequately reflect clinical relevance [66, 67]. Further research is needed to advance more nuanced and standardized methods for assessing meaningful engagement.

Regarding the Information subscale, many MHAs lacked evidence-based content, clear references, thematic coherence, and accessible visual material. This is critical, as the provision of accurate, comprehensible, and evidence-based health information is not only essential for potential effectiveness but also for fostering realistic expectations and, again, sustaining long-term adherence [68]. Additionally, several MHAs displayed shortcomings in app store descriptions, which were sometimes misleading or insufficiently informative. Such shortcomings may not only hinder effective use but also pose safety risks and interfere with appropriate implementation of treatment strategies [41, 68].

The low scores on the Therapeutic Gain subscale underscore an overall limited therapeutic value of the MHAs. Only a minority integrated all three core components of guideline-based obesity treatment [68] and few offered meaningful support for healthcare professionals. While basic psychoeducational content was present in the majority of MHAs, its quality and depth appeared limited, suggesting that its capacity to foster sustainable behavior change might be constrained. These findings are consistent with the results from the Information subscale, where many MHAs lacked high-quality, comprehensive content. Collectively, these limitations raise concerns not only about the suitability of these MHAs as stand-alone interventions but also about their added value within blended care formats. This is particularly important, as blended settings may offer the most promising and realistic context for the implementation of digital tools in obesity treatment [21]. However, it is worth noting that most of the included MHAs were not explicitly designed for such integrated use in routine care—and MHAs that were solely intended for use in blended care were excluded from this review, as their quality could not be reliably assessed.

Evidence on efficacy was found for only two MHAs (“Oviva Direkt” in both app stores). The MHAs have been evaluated through a randomized controlled trial [53] and retrospective analyses [54]. The reported weight loss in the intervention group (−3.2 kg) may seem modest, however, prior evidence indicates that even small reductions in body weight are linked to improvements in blood pressure, fasting glucose, and HbA1c [69–71], highlighting that these effects may still be clinically meaningful. The lack of empirical validation of other MHAs included is in line with findings from previous reviews in other health fields [39, 40, 57, 58] and is likely exacerbated by the mismatch between rapid market turnover and slow-paced academic evaluation [72]. The absence of rigorous scientific testing further raises concerns about the potential benefit of most available MHAs—particularly in light of the low ratings observed on the Information and Therapeutic Gain subscales. As previously stated, the Information subscale explicitly includes an item on the MHA’s evidence base.

Notably, the two versions of “Oviva Direkt,” the only scientifically evaluated MHAs included, also received the highest quality rating in this review (*M* = 4.3 and *M* = 4.2) and were the only ones certified. Although certification frameworks for MHAs differ between countries, they generally involve formal assessment procedures intended to ensure a certain level of safety, quality, and transparency. The present findings therefore suggest that certification may serve as an important mechanism for quality assurance.

### Future directions

The findings of this review, together with the poor correlation between user star ratings and professional assessments observed both here and in previous studies [39, 40, 43, 73], underscore the need for external, evidence-based information platforms (e.g., mhad.science, mindapps.org) that can guide both users and healthcare professionals in identifying high-quality MHAs [46]. However, these platforms are currently often unknown to potential users and therefore underutilized, suggesting a need for increased visibility, dissemination, and integration into routine healthcare pathways.

Beyond evaluation and informed decision-making, future development efforts should specifically target the domains of engagement (including individualization), information quality, and therapeutic potential, which again emerged as critical weaknesses in this review. Promising strategies include the integration of personalized features, blended care elements, and smartphone-specific capabilities such as digital phenotyping [21, 74]. At the same time, future research should prioritize the development of more nuanced and standardized approaches to measuring meaningful engagement in MHAs, alongside more transparent reporting of objective usage indicators of real-world uptake (e.g., retention, completion, frequency of use).

At the same time, future research should prioritize rigorous effectiveness trials of both existing and newly developed MHAs. Ideally, such studies should employ dismantling designs to isolate key mechanisms of action and identify which components drive change.

Moreover, scientific evaluation alone is not sufficient and mechanisms should be implemented to ensure that evidence-based MHAs are actually made available to users in real-world settings [72]. In doing so, it is crucial to ensure equitable access across diverse user groups (e.g., those with limited language skills or complex needs) [21].

Finally, a critical gap remains in the availability of evidence-based MHAs for children and adolescents, despite the early onset and long-term persistence of obesity-related risks [16, 17, 75]. Our findings demonstrate that commercially available MHAs are mostly not designed with pediatric users or their parents in mind. Although evidence on the effectiveness of digital interventions for younger populations is still limited, integrating these age groups into future development and evaluation efforts will be essential to ensure equitable access to effective digital obesity treatment across the lifespan [55, 56].

### Limitations

This study has several limitations. First, it was restricted to MHAs available in German or English and those that did not require external devices, which may have led to the exclusion of MHAs. Second, MHA quality was assessed exclusively using structured expert ratings. Although this approach ensures a standardized and theory-informed evaluation, it does not capture users’ subjective experiences or real-world engagement patterns. While user star ratings were examined in the present study and they did not correlate significantly with overall MARS-G quality scores or any main subscale scores, no systematic user-based evaluation (e.g., structured usability testing or user surveys) was conducted. Third, only one expert rating scale (the MARS-G) was employed, although alternative tools such as ENLIGHT exist [76], and may capture different aspects of MHA quality. Fourth, privacy-related features were assessed based on publicly visible characteristics (e.g., presence of a privacy policy), rather than through technical audits. This may have underestimated actual data security risks, as previously discussed [59]. Fifth, the predefined eligibility criteria led to the exclusion of potentially relevant MHAs, such as those with blended care formats. Sixth, the resulting inclusion of only 21 MHAs limited the sample size and reduced the statistical power for subgroup or correlational analyses. Finally, the rapidly evolving nature of the MHA marketplace poses an inherent challenge to all reviews of this kind, and results may become outdated quickly [77].

## Conclusion

The present quality assessment provides a comprehensive overview of commercially available MHAs for obesity. While they hold promise as scalable tools to support obesity treatment, the current landscape is marked by substantial variability in quality, a lack of scientific validation, and frequent shortcomings in data security and data protection features. To realize the full potential of MHAs in obesity care, coordinated efforts are needed across multiple levels—including the development and dissemination of external, evidence-based evaluation tools, targeted improvements in MHA quality, rigorous effectiveness research, and the translation of validated interventions into routine practice, with particular attention to currently underserved groups such as children and adolescents. Notably, the two highest-rated MHAs in this review were those that had undergone a national certification process (i.e., the German DiGA certification), highlighting the potential value of formal quality assurance and quality management standards for MHAs.

## Supplementary information

Supplementary Material

## Data Availability

The datasets used and analyzed during the current study are available from the corresponding author on reasonable request. Data will only be shared for scientific purposes. Data sharing agreements may have to be signed depending on the request. Support depends on current resources.
