# Supplementary material for: Commercially available mobile health applications for obesity across the lifespan: a systematic search and quality assessment
Source: Int J Obes (Lond). 2026 May 5;50(8):1761–75. doi: 10.1038/s41366-026-02097-0 (PMC13461339; doi:10.1038/s41366-026-02097-0)
Supplement: Supplementary file 1 [file 41366_2026_2097_MOESM1_ESM.docx]

**Supplementary Material**

**Table A. Search terms used.**

| English | German |
| --- | --- |
| obesity | Adipositas |
| overweight | Adipös |
| weight loss | Gewichtszunahme |
| weight control | Übergewicht |
| weight management | Abnehmen |
| corpulence | Gewichtskontrolle |
| fatness | Gewichtsmanagement |
| excessive body fat | Fettleibigkeit |
| bariatrics | Fettsucht |
| weight gain | Starkes Übergewicht |
| body weight disorder | Körperfettüberschuss |
| metabolic syndrome | Metabolisches Syndrom |
| exess fat mass | Erhöhtes Körpergewicht |
| weight reduction | Gewichtsabnahme |
| obese | Dick |
| adiposity | Mehrgewichtig |
|  | Mehrgewicht |
|  | Extremes Übergewicht |
|  | Gewichtsreduktion |
|  | Gewichtsverlust |

**Figure A. Ratings on the subscale therapeutic gain across included MHAs (n = 21)**. The item risk was renamed to security to clarify encoding directions. Ratings lower than 2.5 were categorized as low, ratings between 2.5 and 4 as middle, and ratings of 4 and higher as high
